# EEG-based Brain-Computer Interfaces for people with Disorders of Consciousness: Features and applications. A systematic review

**DOI:** 10.3389/fnhum.2022.1040816

**Published:** 2022-12-05

**Authors:** Valentina Galiotta, Ilaria Quattrociocchi, Mariagrazia D'Ippolito, Francesca Schettini, Pietro Aricò, Stefano Sdoia, Rita Formisano, Febo Cincotti, Donatella Mattia, Angela Riccio

**Affiliations:** ^1^Neuroelectric Imaging and Brain-Computer Interface Laboratory, Fondazione Santa Lucia (IRCCS), Rome, Italy; ^2^Department of Psychology, Sapienza University of Rome, Rome, Italy; ^3^Department of Computer, Control, and Management Engineering “Antonio Ruberti”, Sapienza University of Rome, Rome, Italy; ^4^Servizio di Ausilioteca per la Riabilitazione Assistita con Tecnologia, Fondazione Santa Lucia (IRCCS), Rome, Italy; ^5^Department of Molecular Medicine, Sapienza University of Rome, Rome, Italy; ^6^BrainSigns srl, Rome, Italy; ^7^Neurorehabilitation 2 and Post-Coma Unit, Fondazione Santa Lucia (IRCCS), Rome, Italy

**Keywords:** brain-computer interface (BCI), disorders of consciousness (DoC), EEG, minimally conscious state (MCS), vegetative state (VS), cognitive motor dissociation, Unresponsive Wakefulness Syndrome (UWS), P300

## Abstract

**Background:**

Disorders of Consciousness (DoC) are clinical conditions following a severe acquired brain injury (ABI) characterized by absent or reduced awareness, known as coma, Vegetative State (VS)/Unresponsive Wakefulness Syndrome (VS/UWS), and Minimally Conscious State (MCS). Misdiagnosis rate between VS/UWS and MCS is attested around 40% due to the clinical and behavioral fluctuations of the patients during bedside consciousness assessments. Given the large body of evidence that some patients with DoC possess “covert” awareness, revealed by neuroimaging and neurophysiological techniques, they are candidates for intervention with brain-computer interfaces (BCIs).

**Objectives:**

The aims of the present work are (i) to describe the characteristics of BCI systems based on electroencephalography (EEG) performed on DoC patients, in terms of control signals adopted to control the system, characteristics of the paradigm implemented, classification algorithms and applications (ii) to evaluate the performance of DoC patients with BCI.

**Methods:**

The search was conducted on Pubmed, Web of Science, Scopus and Google Scholar. The PRISMA guidelines were followed in order to collect papers published in english, testing a BCI and including at least one DoC patient.

**Results:**

Among the 527 papers identified with the first run of the search, 27 papers were included in the systematic review. Characteristics of the sample of participants, behavioral assessment, control signals employed to control the BCI, the classification algorithms, the characteristics of the paradigm, the applications and performance of BCI were the data extracted from the study. Control signals employed to operate the BCI were: P300 (*N* = 19), P300 and Steady-State Visual Evoked Potentials (SSVEP; hybrid system, *N* = 4), sensorimotor rhythms (SMRs; *N* = 5) and brain rhythms elicited by an emotional task (*N* = 1), while assessment, communication, prognosis, and rehabilitation were the possible applications of BCI in DoC patients.

**Conclusion:**

Despite the BCI is a promising tool in the management of DoC patients, supporting diagnosis and prognosis evaluation, results are still preliminary, and no definitive conclusions may be drawn; even though neurophysiological methods, such as BCI, are more sensitive to covert cognition, it is suggested to adopt a multimodal approach and a repeated assessment strategy.

## Introduction

Following a severe acquired brain injury (ABI), some patients can show clinical conditions characterized by absent or reduced awareness, known as Vegetative State (VS)/Unresponsive Wakefulness Syndrome (UWS; Laureys et al., 2010), and Minimally Conscious State (MCS), respectively (Bernat, 2006; Laureys et al., 2010). These conditions, along with coma, are known as Disorders of Consciousness (DoC), defined as “prolonged” when a DoC lasts more than 28 days (Giacino et al., 2018; Kondziella et al., 2020).

The comatose state is an acute condition (4 weeks or less) in which a patient lacks both awareness and wakefulness (Bernat, 2006). Coma is typically characterized by the presence of closed eyes with no responsiveness to any kind of stimulation (Bernat, 2006). VS, more recently named UWS (Laureys et al., 2010), is a condition that follows coma, when the patient recovers the vigilance or alertness (eyes opening), but not the awareness of self and surroundings. The patient, in fact, is unable to interact with the environment, in spite of the eyes opening and the recovery of some sleeping-wake cycle (Formisano et al., 2021). Specifically, VS/UWS patients usually demonstrate the following reflexive behaviors: auditory startle response (i.e., a blink, eyelid flutter or any other body startle response, following a loud stimulus), visual startle response (i.e., a blink or eyelid flutter in response to a visual threat presented close to the subject eye), abnormal posturing or withdrawal in response to a nociceptive stimulation, oral reflexive movements (e.g., chewing movements), localization to a sound (i.e., head and/or eyes orient toward the location of the stimulus) (Giacino et al., 2004).

The MCS may follow either coma or VS/UWS as a transient or permanent condition (Beaumont and Kenealy, 2005). It is a condition of severe consciousness alteration in which a minimal but not constant awareness of self and surroundings is present (ability to visually fixate and pursuit, to smile or cry, to localize the painful stimulation, to reach and grasp objects, to follow simple orders in an inconstant and fluctuating way, to verbalize in a sporadic way, to show non-reflexive behaviors) (Giacino et al., 2002, 2004). Indeed, a patient must produce evidence of purposeful and cognitively mediated behavior in a formal clinical assessment to be considered aware. These behaviors are reproducible or long enough to be distinguished from reflexive behaviors (Giacino et al., 2002).

A critical diagnostic marker in DoC is whether or not a patient can follow commands. In fact, while purposeful behavior is considered sufficient to denote awareness of one's external environment, command following indicates, beyond reasonable doubt, that the patient is conscious (Fernández-Espejo and Owen, 2013; Owen, 2013; Gosseries et al., 2014). Command following is a more complex behavior than a non-reflex purposeful movement, since it is intended as the ability to volitionally modulate the behavior in order to produce a coherent response to a request. An example of command following could be the request to look or try to reach one of two objects presented to the patient (from the Coma Recovery Scale – Revised, CRS-R; Giacino et al., 2004). To better distinguish clinical features within the neurobehavioral spectrum (Bruno et al., 2011), the MCS was subsequently divided into two sub-categories: MCS – and MCS + (Bruno et al., 2011, 2012). MCS – patients do not demonstrate evidence of command following; they only show minimal levels of behavioral interaction, characterized by non-reflexive movements: orientation to noxious stimulation, visual pursuit in response to moving stimuli, appropriate movements in response to relevant environmental stimuli (Bruno et al., 2011, 2012). MCS + patients show the ability to follow commands (Bruno et al., 2011, 2012).

Recovery of consciousness or MCS emergence (EMCS) is defined by a behavioral state where the recovery of functional communication or functional object use has been achieved, based on clinical criteria (Giacino et al., 2002; Royal College of Physicians, 2020). Functional locked-in syndrome (fLIS) has been reported as a possible recovery phase from DoC, since the attempts of eye-coded communication shown by the patient are considered as first signs of interaction with the environment, during recovery from VS/UWS or MCS (Formisano et al., 2013). Classical LIS is not a DoC but may be mistaken for one (Childs et al., 1993), and different studies on “covert cognition” or “covert awareness” (Owen et al., 2006; Monti et al., 2010; Bardin et al., 2011; Cruse et al., 2011; Schnakers et al., 2022), revealed that the rate of misdiagnosis of LIS as VS/UWS is actually between 5 and 15% (Vogel et al., 2013; Naci et al., 2017). In the same way, the misdiagnosis rate of persons in MCS as being VS/UWS has been reported to be as high as 43% (Childs et al., 1993; Andrews et al., 1996; Schnakers et al., 2009; van Erp et al., 2015; Wade, 2018), due to the difficulty of performing bedside consciousness assessments of patients with prolonged DoC (Wade, 2018).

The development and use of the CRS-R, assessing the patient's level of consciousness through his/her auditory, visual, motor, oromotor, communication and arousal function (Schnakers et al., 2009) has greatly reduced the rate of clinical misdiagnosis of prolonged DoC (Giacino et al., 2004) and it is considered the gold standard clinical tool (Kondziella et al., 2020). However, a single standard CRS-R behavioral evaluation still leads to a non-zero rate of misdiagnosis, due to the influence of patients' awakening or consciousness fluctuations, movement defects, aphasia (Formisano et al., 2019c), and other problems (Kotchoubey et al., 2014; Cortese et al., 2015). Different studies revealed that repeated behavioral scale evaluations (Wannez et al., 2017), caregiver involvement (Formisano et al., 2019a) and personalized item selection (Stenberg et al., 2018; Sun et al., 2018) of the neurobehavioral-assessment instruments to assess the patient's level of consciousness may improve the reliability of diagnosis.

Functional magnetic resonance (fMRI) (Bardin et al., 2011; Guldenmund et al., 2012; Kondziella et al., 2016) and neurophysiological (e.g., electroencephalography; EEG) paradigms (Cruse et al., 2011, 201; Kotchoubey et al., 2013) using the mental imagery paradigm [i.e., the simulation or re-creation of perceptual experience, across sensory modalities (Kosslyn et al., 2001; Pearson, 2007); e.g., imagine to play tennis or to navigate within your home (Owen et al., 2006; Monti et al., 2010), imagine to open/close the hand (Cruse et al., 2011)] as a channel of communication, are also considered techniques which hopefully could assist in reducing the misdiagnosis rate of patients with DoC (Childs et al., 1993). In fact, these approaches have been used to determine if an unresponsive patient can be “covertly” conscious and can volitionally modulate his/her brain activity, providing evidence of his/her ability to follow commands, a condition defined as “cognitive motor dissociation” (CMD; Schiff, 2015; Schnakers, 2020). Covert awareness is characterized by a dissociation between the inability or extremely limited ability to move, but a preserved cognitive functioning, expressed as a reliable command-following ability detected with neuroimaging or neurophysiological measures. On the contrary, overt awareness is the expression of awareness with overt behavioral responses to external stimuli (Fernández-Espejo and Owen, 2013; Curley et al., 2018). Although it is probably a minor population in terms of numbers, Stender et al. (2014b) espoused that this group of patients, that are functioning in a borderline area between awareness and unconsciousness, represented an important clinical, ethical and societal challenge. In part, this challenge was due to the need to identify covert consciousness in order to facilitate appropriate care and not to create a scenario where such patients would be neglected by caregivers and relatives who assumed there was no conscious awareness present (Kondziella et al., 2016; Sergent et al., 2017).

A Brain-Computer Interface (BCI) is a system that translates brain activity into artificial output, thus modifying the interaction of the central nervous system with the rest of the body or with the external environment (Wolpaw et al., 2020). A BCI may solve different functions (replaces, restores, enhances, supplements, or improves the natural outputs of the brain; Wolpaw et al., 2020) and it has been used with different applications in severely disabled people, such as communication and control (Riccio et al., 2011, 2015, 2022; Schettini et al., 2015), motor and cognitive rehabilitation (Pichiorri et al., 2018; Pichiorri and Mattia, 2020). A BCI has different components: input (i.e., acquisition of brain signals from the user), output (i.e., commands delivered to the system), components that translate input into output (i.e., the signal processing) and a protocol that guides the BCI operation and defines the onset/offset and timing of stimulation, the kind of stimulation necessary to elicit specific brain signals and the feedback delivered to the user. Specifically, the signal processing is a two-step procedure: first signal features, that encode the user's message, are extracted and then an algorithm translates these features into device command orders that carry out the user's intent (i.e., *classification procedure*; Wolpaw et al., 2002).

A BCI can be intended as an active or a passive system. The active BCIs require an active modulation of user's brain activity through an engagement in a specific task (e.g., to count the rare stimuli or own name, to imagine a movement). The passive BCI can passively decode mental, emotional, and cognitive states from the neurophysiological signals of the user. These systems do not require any active modulation of user's brain activity or engagement in a specific task, as in the active BCI (Zander et al., 2009; Han et al., 2022; Sciaraffa et al., 2022). Finally, the BCI also includes hybrid systems (h-BCI), which integrate two different brain signals to produce its output or which combine a BCI output with a muscle-based one (Choi et al., 2017).

Given the large body of evidence that some patients with DoC possess “covert” awareness (Owen et al., 2006; Boly et al., 2007; Monti et al., 2010; Bardin et al., 2011; Fernández-Espejo and Owen, 2013; Stender et al., 2014a; Schnakers et al., 2022), they are candidates for intervention with BCI. The first attempt to establish an alternative communication channel with DoC patients, independent from motor behaviors and overt consciousness, was made by Monti et al. (2010). Based on previous results from Owen et al. (2006), they proposed a mental imagery fMRI paradigm to assess the ability of command-following in 54 patients with DoC. They found that 5 patients (2 VS/UWS) could intentionally control their mental activity. Furthermore, one MCS patient was tested for communication with this paradigm: the 2 mental imagery tasks were coupled with the “yes” and “no” answers and he managed to answer 5 out of 6 autobiographical questions with this method. The presence of distinct neural activations in response to two different mental imagery tasks, comparable to the healthy controls, allowed to infer that the patients decided to cooperate with the experiment, and this could be interpreted as a clear act of intention, reflecting awareness of themselves and their surroundings (Owen et al., 2006).

In this regard, BCI development for patients with DoC can fulfill important clinical diagnostic functions, such as cognitive assessment and awareness detection, supporting the behavioral diagnosis (Gibson et al., 2016; Annen et al., 2020a). In a rehabilitation context, BCI methods reflecting the preservation of at least minimal consciousness can identify a purposeful external behavior or ability to covertly follow commands (i.e., the ability to execute a mental imagery task or an active counting task in order to control a BCI), helping clinicians and researchers to focus their efforts on training the patient to employ this purposeful behavior for functional communication, with potential diagnostic benefits and significant ethical implications (Formisano and Zasler, 2020).

The brain signals usually adopted with DoC patients to implement a BCI are: the P300, both alone and in combination with the Steady State Visual Evoked Potentials (SSVEPs), resulting in a h-BCI, the sensorimotor rhythms (SMRs) and other modulations related to the power spectrum. Some differences have been observed in the amplitude and latency of the P300 between DoC patients and healthy subjects and among different DoC diagnosis. Indeed, it is possible to observe a larger P300 amplitude in healthy controls with respect to DoC patients and a larger P300 amplitude in MCS patients with respect to the VS/UW patients, especially when comparing active vs. passive tasks (Schnakers et al., 2008; Risetti et al., 2013). However, the P300 amplitude is not always sufficient to discriminate among different DoC diagnosis (Real et al., 2016). Furthermore, DoC patients frequently had a delayed P300 latency with respect to healthy controls (Perrin et al., 2006; Sergent et al., 2017). Since VS/UWS patients should not be able to modulate their real or imagined behavior, SMRs should not be present in these patients in response to a motor command; however some studies found that some VS/UWS patients were able to modulate their SMRs (Formaggio et al., 2020). It should be possible to observe a modulation in the SMRs in MCS patients in response to a command requiring real or imagined movements (Owen et al., 2006; Monti et al., 2010; Cruse et al., 2011). Finally, the spectral power is characterized by typical modulations related to the continuum of consciousness (Formisano et al., 2011). Indeed, it seems that a diagnosis of DoC is associated with a decrease in the activity of the high frequency bands (alpha and beta bands) and with an increase in the low frequency bands (delta and theta bands) with respect to healthy subjects (Lechinger et al., 2013; Stefan et al., 2018).

We conducted a systematic review to evaluate the current state of BCI research with DoC patients. Although many reviews have been published about BCI and DoC, these are mainly narrative and non-systematic overviews about the advances in the field of the neurophysiology of DoC, including the BCI as one of the neurophysiological tools available to support the assessment of consciousness (Kübler and Kotchoubey, 2007; Kübler, 2009; Chatelle et al., 2012; Naci et al., 2012; Mikołajewska and Mikołajewski, 2014; Luauté et al., 2015; Gibson et al., 2016; Li et al., 2016; Annen et al., 2020a; Comanducci et al., 2020; He et al., 2022; Xu et al., 2022). Therefore, the present work is specifically focused on studies testing a BCI on a sample of participants including at least one DoC patient. We considered EEG-based systems, due to their portability, affordability, safety and ease of use; these characteristics make the EEG the most suitable instrument to measure brain activity in DoC patients, whose physical and medical conditions may prevent from other measurements such as fMRI (Formisano et al., 2019b). We aimed at: (i) describing the characteristics of the EEG-based BCI systems developed for DoC patients, in terms of control signals adopted to operate the system, characteristics of the paradigm, classification algorithms and applications; (ii) evaluate the performance of DoC patients with a BCI.

## Methods

The present systematic review followed the Preferred Reporting Items for Systematic Reviews and Meta-Analyses guidelines (PRISMA; Page et al., 2021) to search and extract eligible studies.

### Search

We adapted the search strategy using the Population, Intervention, Comparison, Outcome (PICO) framework without specifying the type of “Comparison”. We considered DoC as target “Population”, BCI as the “Intervention” and the BCI performance as the “Outcome”. The main review question was: *Which are the characteristics and applications of BCI in DoC patients, and which is the performance of DoC patients with a BCI?*

The search and selection of papers were performed through the following databases: PubMed, Scopus, Web of Science and Google Scholar. The relevant papers were collected using the following keywords: {[brain-computer interface] AND [(disorders of consciousness) OR (minimally conscious state) OR (vegetative state) OR (unresponsive wakefulness syndrome) OR (coma) OR (cognitive motor dissociation)]}. The results obtained from each database were exported to a web-based bibliographic management software (i.e., Mendeley; https://www.mendeley.com. Last access: 30th of September 2022) to merge all research results and remove duplicates.

### Eligibility criteria

Studies were considered eligible if they met the following inclusion criteria: (i) enrollment of at least one patient with DoC, meaning a comatose patient, a patient in VS/UWS or in MCS; (ii) testing an EEG-based BCI system (intended as a system with a classification procedure); (iii) studies published in international peer-review journals; (iv) research articles; (v) studies published in English. Articles were excluded if they met the following exclusion criteria: (i) studies enrolling only patients with LIS or in EMCS; (ii) abstracts, reviews, editorials, letters, notes, and conference papers reporting preliminary results successively presented in a journal article.

### Screening

After the deletion of duplicate papers, articles were first screened by reading titles and abstracts, so that all articles not matching the aim of the review and the inclusion/exclusion criteria were excluded. The full text of all the papers included after the first screening was read and assessed for eligibility according to the inclusion/exclusion aforementioned criteria.

### Data extraction

According to the aims of the review, the following data were extracted from each selected article and reported in Supplementary Table 1: (i) information about the sample of participants involved in each study in terms of number of healthy and DoC participants, diagnosis of DoC participants and behavioral scale adopted to define it; (ii) BCI application (i.e., assessment, communication, prognosis and rehabilitation); (iii) BCI control signals; (iv) number and position of the EEG channels; (v) classification algorithm used; (vi) characteristics of the paradigm: information about the tasks (i.e., oddball, motor imagery, motor action) and the timing and number of stimuli delivered; (vii) BCI performance, summarizing the main results of each study, providing information in terms of patients that controlled the BCI and the definition of the chance level, when available.

## Results

### Search

**Figure 1:**
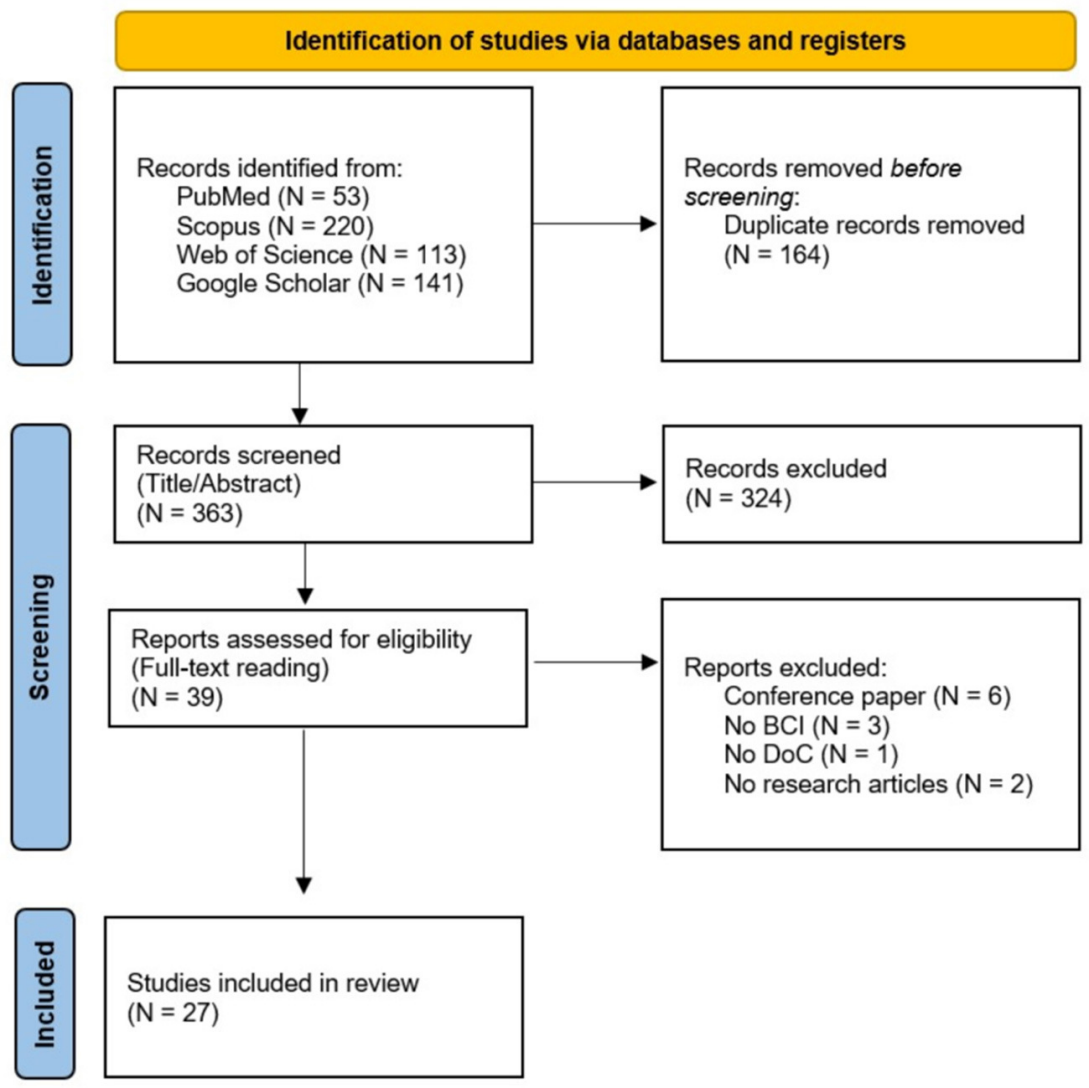
PRISMA flow diagram of the selected studies.

The first run of the search identified a total of 527 results (Scopus: 220 results; Web of Science: 113 results; PubMed: 53 results; Google Scholar: 141). After the automated deletion of duplicates, 363 results were identified. The first screening by title and abstract identified 39 papers and after the full-text reading, 27 articles were included in the final database (see Figure 1; PRISMA flow diagram). After the full-text reading, articles were excluded because:

- they were conference papers reporting preliminary results successively presented in a journal article (*N* = 6);
- no BCI was tested (*N* = 3);
- no DoC patients were included in the paper (*N* = 1);
- they were no research papers (e.g., tutorial articles; *N* = 2).

Articles included were published from September 2013 to August 2022.

### Characteristics of included studies

The Supplementary Table 1 summarizes all the characteristics extracted from the studies, which are extensively described in the following sections.

#### Sample of participants and behavioral assessment

The total number of patients with DoC included in the studies was 390 (considering 26 out of the total 27 studies): 4 patients were in coma, 195 were VS/UWS patients and 186 were MCS patients. The number varied from a minimum of 4 patients (Coyle et al., 2015) to a maximum of 78 DoC patients (Pan et al., 2020), with an average (±standard deviation, SD) of 15 (±14.5) DoC patients for paper (not considering LIS and EMCS patients included in the papers). Four studies had a sample of participants including 20 or more patients (Xiao et al., 2016; Annen et al., 2020b; Murovec et al., 2020; Pan et al., 2020). The total number of patients and the average do not include data from Eliseyev et al. (2021). They did not specify the diagnosis of the DoC patients involved in the paper, but they divided the sample (*n* = 18) into conscious/unconscious (*n* = 14/4) patients according to the Command Following Score (CFS; Claassen et al., 2016, 2019). Furthermore, Guger et al. (2017) did not specify the diagnosis of the DoC patients. Therefore, the amount of VS/UWS and MCS patients previously mentioned does not consider data from these two studies. Twenty-three studies (85.2%) included both patients with a diagnosis of VS/UWS and MCS. Pokorny et al. (2013) and Coyle et al. (2015) described only patients with MCS; Guger et al. (2018) and Spataro et al. (2018) included only patients in VS/UWS. Twenty-one (77.8%) studies included a control group of healthy subjects (no control group: Coyle et al., 2015; Guger et al., 2017, 2018; Wang et al., 2017; Annen et al., 2018; Spataro et al., 2022). Furthermore, 11 (40.7%) studies also included LIS and EMCS patients (see Supplementary Table 1).

In 25 studies (92.6%) the diagnosis of the participants was established with the CRS-R (Giacino et al., 2004). One study (Eliseyev et al., 2021) administered the CFS to classify patients into conscious and unconscious, which assesses the ability of patients to follow a verbal command with a motor response. Finally, one study (Guger et al., 2017) did not specify the behavioral scale adopted to define the diagnosis (see Supplementary Table 1).

#### Control signals and EEG channels

Four control signals were identified to control the BCI (see Supplementary Table 1; Table 1):

- P300 (*N* = 19; 70.4%). The P300 is an Even-Related potential (ERP) component which is generated when a salient or a rare stimulus is presented; it is independent from the sensory modality selected to deliver the stimulation (Polich, 2007);
- hybrid control signals (h-BCI; *N* = 4, 14.8%). In all the papers included in the present review, the h-BCI is intended as a system integrating two different brain signals to produce an output. Especially, the signals integrated in these studies were the aforementioned P300 and SSVEPs. The SSVEP is a periodic response evoked by a visual periodic stimulation (Norcia et al., 2015);
- SMRs (*N* = 5, 18.5%). SMRs refer to oscillations in the mu (8–12 Hz) and beta bands (13–30 Hz), associated to real or imagined movements; especially, movements or preparation to movement are associated to a decrease in mu and beta rhythms (event-related desynchronization; ERD), while relaxation is associated to increase in the same rhythms (event-related synchronization; ERS) (Wolpaw et al., 2002);
- brain rhythms elicited by an emotional task (i.e., power spectrum; *N* = 1, 3.7%). This paper refers to spectral power changes induced by emotional stimuli and specifically considers the differential entropy as a feature (Huang et al., 2021a).

**Table 1:** The table presents the number of studies which employed a specific control signal for each application (within each cell).

	**P300 ERP**	**h-BCI (P300, SSVEP)**	**SMRs**	**Power spectra**	**Total “Applications” *n* (%)**
Assessment	19	3	4	1	26 (96.3%)
Communication	6	1	2	–	7 (25.9%)
Prognosis	1	1	–	–	2 (7.4%)
Rehabilitation	1	–	–	–	1 (3.7%)
Total “Control signals” *n* (%)	19 (70.4%)	4 (14.8%)	5 (18.5%)	1 (3.7%)	

The final row “Total control signals” reports the number (and percentage with respect to the total number of studies included) of studies employing each control signals. The final column “Total Applications” reports the number (and percentage with respect to the total number of studies included) of studies testing each application. Due to an overlap of different control signals and applications within some papers, the two totals reported do not correspond to the sum of the cells of the table.

The specificity of some paradigms produced other ERP components, apart from the P300, which were considered relevant in the analysis. Specifically:

- In Xiao et al. (2018b) two additional relevant ERP components were considered: the N170 and the motion-onset VEP. The N170 is a face-sensitive potential and the motion-onset VEP is a potential composed of 3 components (P100, N200, P200) sensitive to the presentation of visual moving stimuli. These potentials were coherent with the Graphical User Interface which was based on the presentation of moving faces;
- In Xie et al. (2018) two additional relevant ERP components were considered: the N400 and the Late Positive Component (LPC). Different results about these two components are reported, since they were not found in all the DoC patients and they did not manage to differentiate among VS/UWS and MCS patients (see Wutzl et al., 2021 for a review). These two components are related to the semantic processing, since the paradigm required the participant to discriminate between stimuli semantically congruent/incongruent;
- In Xiao et al. (2016, 2022), the Mismatch Negativity (MMN) was considered in addition to the P300. The MMN is a negative component generated in response to the violation of a rule. The MMN in DoC patients has been associated with different results; it is able to distinguish between healthy and DoC patients, but not between different DoC diagnosis (see Wutzl et al., 2021 for a review).

The number of channels varied from 3 to 32: from 8 to 32 channels were used for both the P300-based and sensorimotor-based BCIs, from 9 to 30 channels were acquired for the hybrid BCI and 32 channels were acquired for the brain rhythms elicited by emotional tasks (see Supplementary Table 1).

#### Applications

Four BCI-applications were identified (see Supplementary Table 1; Table 1):

- *Assessment of consciousness* (*N* = 26; 96.3%): the studies investigated the ability of command following, by means of different kind of active tasks: counting the occurrence of a target stimulus in a classical oddball paradigm, processing numerical information, recognizing emotions, and accomplishing a task from the CRS-R, transposed in a BCI customized interface. Six studies aimed at evaluating the ability of patients to accomplish tasks from the CRS-R with a BCI. The items transposed in a BCI interface were the following: the visual fixation item (Xiao et al., 2018a), the visual pursuit item (Xiao et al., 2018b), the object recognition item (Wang et al., 2019), the auditory startle item (Xiao et al., 2016), the sound localization item (Xiao et al., 2022) and the communication item (Wang et al., 2017). All these studies implemented a BCI relying on the P300.
- *Communication* (*N* = 7; 25.9%): the studies implemented BCI interfaces to support communication.
- *Prognosis* (*N* = 2; 7.4%): the studies evaluated the relation between the BCI performance and the clinical outcome (Spataro et al., 2018; Pan et al., 2020).
- *Rehabilitation* (*N* = 1; 3.7%): a single study (Murovec et al., 2020) that evaluated the effects of a training with a P300-based vibrotactile BCI on the level of consciousness.

#### Classification algorithms

Regarding the classification algorithms, the Support Vector Machine (SVM) classifier is the most frequent algorithm applied to classify EEG features (*N* = 14 papers; 51.9%), followed by the Linear Discriminant Analysis (LDA; *N* = 9, 33.3%). Two studies used the Stepwise Linear Discriminant Analysis (SWLDA; Lulé et al., 2013; Pokorny et al., 2013), Eliseyev et al. (2021) applied the Recursive Exponentially Weighted N-way Partial Least Squares (REW-NPLS) and Xiao et al. (2016) proposed a new “peak detection algorithm”. Finally, Höller et al. (2013) compared different classification methods to investigate the most appropriate ones for DoC patients. Specifically, they compared the DADF (discriminant analysis with diagonal quadratic function), the knn (k-nearest neighbor) and the SVM (see Supplementary Table 1).

#### Paradigms

**Figure 2:**
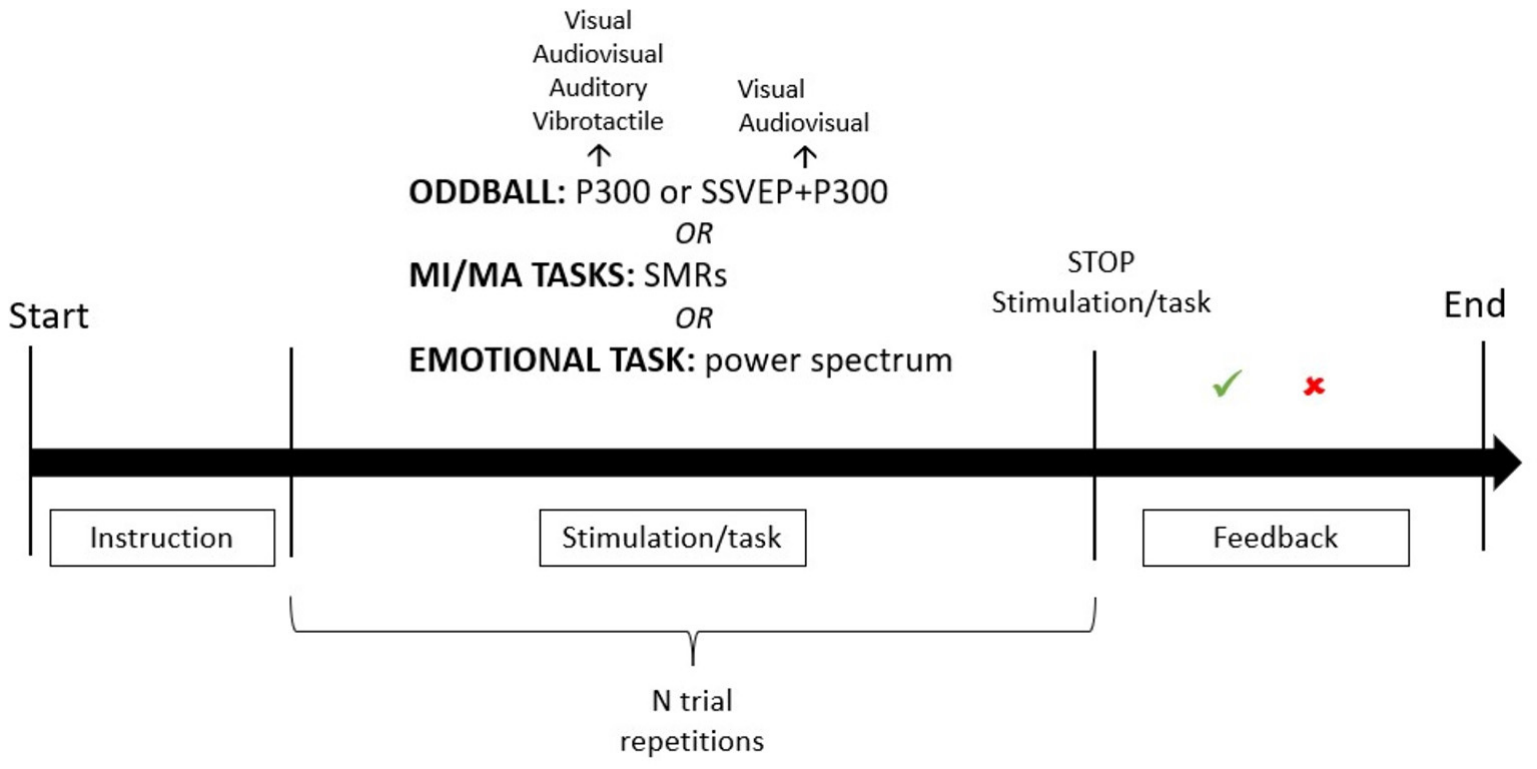
General representation of a BCI paradigm. The figure illustrates a general pipeline of a BCI paradigm which could be adapted to each study included in the review. First, an instruction about the task is presented. After the instruction, the task starts and the stimulation is delivered. The possible tasks are: the oddball task, the motor imagery/motor action task or the emotional task. The oddball task is used both in the P300-based BCI and in the h-BCI; it could be presented in different sensory modalities (visual, audiovisual, vibrotactile and auditory for the P300-only BCI; visual or audiovisual for the h-BCI). A different number of trial repetitions can be delivered according to the specificity of each paradigm. After the stimuli presentation, the stimulation is interrupted, and the system delivers feedback about the appropriateness of the user's response. A period of rest usually follows the feedback. See Supplementary Table 1 for further information about the timing of the stimulation, the number of trial necessary to each paradigm, the number of stimuli adopted in the interface/stimulation protocol.

The typology of tasks proposed is recurrent according to the EEG control signal considered (see Figure 2 for a general pipeline). The most frequent task is the oddball (*N* = 23; 85.2%) to elicit the P300. The oddball paradigm consists in the presentation of a series of at least two kinds of stimuli, a target (rare) one and a non-target (frequent) one; the presentation of the target stimulus elicits the P300. It was employed in various forms of sensory stimulation: vibrotactile (29.6%), visual (22.2%), auditory (22.2%), and audiovisual (25.9%). Vibrotactile active oddball was the most frequent (*N* = 8) sensory modality adopted to deliver the stimulation, and it was based on sequences of 2 or 3 stimuli. Visual and auditory oddballs were present in 6 studies each, always in active mode, except for 1 study that used a passive mode (Xiao et al., 2016). The active mode consisted in the active engagement of the subject in the task proposed; the participant was asked to attend and mentally count the occurrence of a target stimulus (in the oddball paradigm) or to perform a (mental) motor task. In the case of the passive mode, the participant was exposed to the stimulation without specific instructions. The auditory oddball was presented as a sequence of 2 stimuli (a standard one vs. a deviant one) in all the studies except for one (Lulé et al., 2013), which proposed a sequence of 4 stimuli. The visual modality was also employed in the h-BCI and consisted of 2 stimuli simultaneously flickering and flashing, which elicited both the P300 and the SSVEPs. Two single studies (Xiao et al., 2018a,b) proposed a visual-only P300-based BCI, presenting 4 images. Audiovisual oddball is used in 7 studies. In all the studies except for Pan et al. (2018), who used emotional videoclips as audiovisual stimuli in an oddball paradigm, the audiovisual stimulation consisted in the presentation of a visual oddball interface with a simultaneous auditory stimulus which can be congruent or not with the visual stimulus. The studies testing visual and audiovisual BCIs differed in terms of the kind of stimuli presented in the interface, that the subject had to attend (e.g., numbers, photos, etc.; see Supplementary Table 1).

SMRs were elicited by motor action (MA) and motor imagery tasks (MI). The motor action task characterized 1 study (3.7%), in which the participant was asked to open/close the right hand (Eliseyev et al., 2021). Motor imagery tasks were used in 4 works (14.8%): 3 of them (Höller et al., 2013; Guger et al., 2017; Chatelle et al., 2018) asked the participants to imagine opening/closing the hand; Coyle et al. (2015) asked participants to imagine moving the hand or the foot toe. Finally, one study (Huang et al., 2021a), developed a passive emotion recognition paradigm to elicit specific brain rhythms.

The column “Stimulation” in Supplementary Table 1 reports further information about the number of stimuli delivered and the timing of the stimulation for each paradigm.

#### BCI performance

In this section, we reported the main results of the studies included, in terms of number of DoC patients obtaining a significant classification accuracy (i.e., classification accuracy statistically above the chance level). Pokorny et al. (2013), who tested an auditory P300-based BCI, was the only study that did not obtain any significant result.

Regarding the group of studies testing a P300-based BCI for CRS assessment, all of them, showed a proportion (range: 30.8–78.6%) of patients reaching a significant classification accuracy, indicative of the ability to accomplish a specific CRS task, even if they did not show the corresponding behavioral response in the CRS-based assessment. Xiao et al. (2018a) found that 3 (2 MCS, 1 LIS) out of 4 patients (30.8%) who had a classification accuracy above the chance level, showed a coherent behavioral response to the corresponding CRS-R item (i.e., visual fixation), while the remaining VS/UWS patients did not show a behavioral visual fixation. However, in 2 patients (1 MCS, 1 EMCS; 15.4%) a dissociation was found between a consistent response to the behavioral assessment and the absence of the corresponding response to the BCI assessment. In a successive study, Xiao et al. (2018b) found that 11 of the 14 patients tested (78.6%) showed “visual pursuit” in the BCI assessment: 4 patients (2 MCS, 1 EMCS, 1 LIS) were classified as responsive by both BCI and CRS-R assessment, while 7 (4 VS/UWS, 3 MCS) patients showed visual pursuit only in the BCI assessment. However, the visual pursuit was observed behaviorally in 5 (3 VS/UWS, 2 MCS) of these 7 patients in the 2-month follow-up CRS-R assessment. In Wang et al. (2019), 6 of the 13 patients tested (2 VS/UWS, 3 MCS, 1 LIS; 46.2%) obtained a classification accuracy above the chance level in the “object recognition” item without showing a coherent score in the behavioral CRS-R; furthermore, 3 of them showed object recognition at the follow-up CRS-R 2 months later. Xiao et al. (2016) detected the auditory startle response with a BCI in a passive mode in 14 (9 VS/UWS, 4 MCS, 1 EMCS; 73.7%) of the 19 patients tested; three of them, diagnosed as VS/UWS, did not show such response in the behavioral assessment. In a successive study, Xiao et al. (2022) investigated the “sound localization” item. They found that 11 patients (5 VS/UWS, 6 MCS; 61.1%) had a significant classification accuracy in the BCI assessment: 4 MCS patients reached the score corresponding to the “sound localization” item in the CRS-based assessment, while 7 patients did not reach the sound localization score in the behavioral assessment. Finally, in Wang et al. (2017), 8 of the 13 patients tested (4 VS/UWS, 4 MCS; 61.5%) had a significant classification accuracy: one MCS patient was responsive to both the CRS-R and BCI assessment, while 7 patients who achieved a significant classification accuracy in the BCI assessment, did not reach the corresponding CRS-R score in the communication subscale.

A series of studies (*N* = 8) used vibrotactile, with 2 or 3 stimuli, and binary auditory paradigms to assess the presence of consciousness, with the final aim to use the vibrotactile BCI to test communication (i.e., “*mindBEAGLE*”; Allison et al., 2017). Spataro et al. (2018) reported that only 3 out of 12 VS/UWS patients tested (25%) obtained a significant classification accuracy in the vibrotactile paradigms. They found a correlation between the classification accuracy and the 6-months follow-up CRS-R score; the same correlation was not found with the CRS-R score at the moment of the experiment. Thus, they concluded that performance in these paradigms might be a clinical marker of recovery. Spataro et al. (2022) also conducted a study to investigate the efficiency of the vibrotactile BCI to detect command following with respect to the CRS-R, across multiple sessions. They found that the BCI could detect command following before the CRS-R in 7 out of the 16 patients tested. Furthermore, 4 behaviorally unresponsive patients showed neurophysiological signs of command following. Guger et al. (2018) tested 12 VS/UWS patients and found that 7 (58.3%) and 5 (41.7%) patients had a discriminable brain response to target stimuli in the vibrotactile paradigm with 2 and 3 stimuli, respectively. Furthermore, 2 VS/UWS patients, who obtained a classification accuracy above 70% in the vibrotactile paradigm with 3 stimuli, were tested for communication: the first one answered correctly to 4/5 questions and the second one answered correctly to 6/10 questions and 7/10 questions, in 2 separate sessions. Annen et al. (2018) reported that only one MCS of 12 patients involved reached a classification accuracy above 70% in the vibrotactile paradigm with 2 stimuli; he was subsequently tested with the vibrotactile paradigm with 3 stimuli. Even if he obtained a classification accuracy of 70% during the assessment, he did not manage to use the BCI for functional communication, since he answered correctly to 1 out of 6 autobiographical questions. Murovec et al. (2020) found that the classification accuracy with a vibrotactile BCI, based on 3 stimuli, was above the chance level in 10 out of 20 DoC patients (50%; 11 VS/UWS, 9 MCS) in the first session of a repeated assessment, whereas every patient reached a significant classification accuracy in his best run. Furthermore, they found a significant difference between the CRS-R score before and after the training in all the patients. Chatelle et al. (2018) found that 3 out of 9 acute DoC patients tested (33.3%; 2 coma, 1 VS/UWS) reached a significant classification accuracy in the auditory BCI assessment, whereas 2 DoC patients (22.2%; 1 VS/UWS, 1 MCS) and 1 LIS achieved a classification accuracy above the chance level in the vibrotactile paradigm. Furthermore, they tested the LIS patient for communication, since he obtained a classification accuracy above 60% during the assessment; he managed to answer correctly one out of 2 questions. They also tested a MI-based BCI, but none of the patients obtained a significant classification accuracy. Annen et al. (2020b) pointed out that about a quarter of the patients tested showed a response to the stimulation only to one of the two paradigms proposed (binary auditory oddball, binary vibrotactile oddball), demonstrating the importance of a multimodal assessment. Finally, Guger et al. (2017) found that 7 out of the 8 patients tested (5 DoC, 3 LIS) reached a significant classification accuracy higher than 80% with a least one of the paradigms tested (auditory or vibrotactile, MI). Furthermore, they tested the communication and they found that 3 patients (1 DoC, 2 LIS) were able to efficiently communicate with the vibrotactile BCI and only 1 LIS patient managed to use the MI-based BCI for communication; these patients managed to answer at least 4 out of the 5 questions proposed during the communication session.

Within this group of studies testing a P300-based BCI, it is possible to include the following studies that implemented different oddball paradigms with respect to the previously described ones. Lulé et al. (2013) tested 18 patients with an auditory 4-choice P300-based BCI and they found that only one MCS patient and one LIS patient reached a significant classification accuracy. The MCS patient did not show any sign of command following at the CRS-R assessment. However, only the LIS patient reached a significant communication rate (60%). Two studies (Wang et al., 2015; Xie et al., 2018) tested audiovisual BCIs based on the ability to process numerical information. They found a significant classification accuracy in 5 out (71.4%; 1 VS/UWS, 4 MCS) of 7 patients tested (Wang et al., 2015) and in 3 out of 8 patients tested (1 VS/UWS, 2 MCS; 37.5%) (Xie et al., 2018).

Pan et al. (2018) and Huang et al. (2021a) developed a visual BCI aiming at testing emotion recognition as a way to assess consciousness, the first one based on the P300 and the second one based on the power spectrum. Pan et al. (2018) found that 3 of the 8 patients tested (37.5%; 1 VS/UWS, 2 MCS) obtained a significant online accuracy and in 2 patients (1 VS/UWS, 1 MCS) this result was also confirmed by a significant accuracy in the offline spectral analysis. Huang et al. (2021a) found that 3 (2 MCS, 1 EMCS; 37.5%) out of 8 patients tested obtained an online accuracy significantly higher than the chance level.

A group of studies (*N* = 4) tested h-BCIs based on the combination of the P300 and the SSVEPs. Among these studies, Pan et al. (2014) found that 5 of the 8 patients tested (2 VS/UWS, 2 MCS, 1 LIS; 62.5%) reached a significant classification accuracy in the first run and 3 (1 VS/UWS, 1 MCS; 1 LIS) out of these 5 patients reached a significant classification accuracy also in the second and third run. In a successive study, the authors (Pan et al., 2020) implemented 3 paradigms based on different stimuli: a visual photograph paradigm, a visual number paradigm and an audiovisual number paradigm. They aimed at investigating the presence of cognitive motor dissociation (CMD) and if BCI performance could be a discriminant marker for prognosis evaluation by comparing CRS-R scores between the moment of the BCI sessions and 3 months later. According to their BCI performance, patients were divided into CMD and potential non-CMD. Results showed that 44% of the 78 patients involved (45 VS/UWS, 33 MCS) could be defined as patients with CMD, since they had a significant BCI accuracy in at least one paradigm. They also found a significant correlation between BCI accuracies and the clinical outcome: statistically, the CMD patients had a better outcome than potential non-CMD patients. Li et al. (2015) proposed 3 tasks of mental arithmetic to assess awareness. Five of the 11 patients enrolled (2 VS/UWS, 2 MCS, 1 EMCS; 45.5%) reached a significant classification accuracy both in a task of number recognition and number comparison and 3 out of 5 patients (1 VS/UWS, 1 MCS, 1 EMCS) reached a significant classification accuracy also in a mental calculation task. Finally, Huang et al. (2021b) implemented a hybrid asynchronous BCI as a communication channel and 3 MCS patients out of 7 DoC patients tested (42.9%) gained accuracies higher than the chance level.

Eventually, with respect to the studies testing a BCI based on sensorimotor rhythms, Höller et al. (2013) proposed a motor imagery task to examine a set of 20 features and 3 classification methods (DADF, knn, SVM), with the aim to identify which resulted in the best accuracy in healthy subjects and then to transpose results on DoC patients. The study involved 22 healthy subjects and 14 patients with DoC (9 VS/UWS, 5 MCS). Coherences showed the best reliability among healthy subjects and VS/UWS and MCS groups, although feature extraction and classification in patients don't provide a validation ground for results. Results from Coyle et al. (2015) suggested that a MI-based BCI systems can complement awareness assessment tests (4 MCS patients tested). Finally, Eliseyev et al. (2021), who tested BCI based on a motor action paradigm, obtained significant results only in 5 of the 14 conscious patients tested, while none of the 4 unconscious patients reached a significant accuracy.

## Discussion

### Patients and behavioral assessment

The aim of the present work was to systematically review the literature about BCI in the field of DoC, in order to describe the characteristics and applications of the EEG-based BCI systems developed for DoCs and the performance of BCI in DoC patients. We identified 27 studies published from 2013 to 2022, that reported about BCI systems in patients with DoC. The studies included a total of 390 patients with DoC, most frequently including both patients in VS/UWS and patients in MCS (85.2%). A total of 4 patients in coma, 195 patients in VS/UWS and 186 patients in MCS were involved (min: 4; max: 78). The number of patients included in the studies was highly variable, as showed by the standard deviation of ±14.5 patients with an average of 15 patients. The high variability is mainly due to the presence of a small sample size in most of the studies; only 2 studies had a sample of participants including more than 30 patients (Annen et al., 2020b; Pan et al., 2020) and 2 studies had a sample of 20 subjects (Xiao et al., 2016; Murovec et al., 2020). The presence of such a small sample size in BCI research in the DoC field, may be due to several factors, such as the precarity of DoC patients' medical condition and the presence of comorbidities (Pistoia et al., 2015; Estraneo et al., 2018), which influence the possibility to include patients in a research protocol and to complete the experimentation. The issues deriving from a small sample size, as for instance the high variability of results, lead us to underline the need for further research including larger samples of patients. In the 92.6% of the studies the diagnosis of the participants was defined based on the CRS-R, confirming a coherence in the instrument used for the behavioral assessment. However, most of the studies performed such assessment only one time (mostly at the beginning of the inclusion process) or they did not specify if the diagnosis was based on more than one behavioral assessment. This is a relevant aspect, since it was previously demonstrated the need of performing at least five assessments in each patient with disorder of consciousness to reduce misdiagnosis (Wannez et al., 2017). However, seven studies clarified that they based the assessment process on a repeated CRS-R assessment, following the guidelines from Wannez et al. (2017) (Annen et al., 2018, 2020b; Guger et al., 2018; Spataro et al., 2018; Xiao et al., 2018a; Xie et al., 2018). The importance of following the gold standard guidelines in the behavioral assessment is essential to guarantee a correct interpretation and comparison of results with the BCI.

### Control signals

In the majority of the studies included, the EEG-based BCIs were based on the P300, both alone (70.4%) and in combination with the SSVEPs, resulting in a hybrid control (14.8%). These data show that the P300-based BCIs are the most investigated in the field of DoC and the oddball paradigm is the most frequent task adopted, as one of the paradigms traditionally used to elicit the P300.

Furthermore, the P300 was elicited by paradigms built in different sensory modalities: vibrotactile (29.6%), visual (22.2%), auditory (22.2%), and audiovisual (25.9%). The audiovisual modality is one of the most frequently adopted; this may be explained with the need of a multisensorial approach in this target population, since the variety of lesions characterizing DoC patients may compromise different sensory pathways and cerebral networks (Annen et al., 2020a). Annen et al. (2020b), who specifically addressed the issue of a multimodal assessment, found that about a quarter of the patients had a significant response to one sensory modality only, supporting the necessity of a system taking advantage of different sensory channels. A similar result was previously reported in Schreuder et al. (2013), who found a dissociation in the performance of a severe brain injured patient between the auditory and the visual modes of a P300-based BCI. Indeed, it is mandatory to disambiguate if the lack of results is due to the absence of consciousness or to the disruption of a specific sensory pathway. The need for a multisensorial approach may also account for the similar percentage of BCIs in the different sensory modalities: there is not a prevalent sensory channel employed among the different studies included. It is crucial to adapt the system to the characteristics of the patient.

Only the 18.5% of the studies used a SMR-based BCI and one study proposed a BCI employing the power spectrum as control feature, underlining the need for further investigation in these areas. SMR-based BCIs obtained more variable results; indeed, Coyle et al. (2015) stated the role of BCI in supporting the diagnosis, and Eliseyev et al. (2021) found some significant results in 5 of the 18 patients tested, even if they mainly obtained these results on conscious patients, without further specifying the diagnosis of the patients. On the contrary, Höller et al. (2013) and Chatelle et al. (2018) could not find significant results with a SMR-based BCI. Regarding the power spectrum as control feature, the authors (Huang et al., 2021a) obtained some interesting results, since 37.5% of the patients showed the ability to correctly recognize emotions, which can be interpreted as an expression of consciousness.

### Applications of BCI

The most widespread application of BCI is the assessment of consciousness (96.3%). Indeed, the high misdiagnosis rate with behavioral assessment scales draws attention on the importance of alternative methods to detect covert consciousness (Wade, 2018). The studies from Owen et al. (2006) and Monti et al. (2010), focused on the detection of covert consciousness in behaviorally unresponsive patients with neuroimaging methods, opened the way to several studies using both fMRI and EEG paradigms with this aim [i.e., command following, mental imagery and oddball paradigms (Schnakers et al., 2015b; Kondziella et al., 2016; Schnakers, 2020)]. Moreover, the assessment of consciousness is considered a mandatory issue to address, before implementing any other BCI application (e.g., communication) with DoC patients. The characteristic of BCI to directly measure brain activity while carrying out a task, without relying on muscles or peripheral nerves, makes it a promising tool in supporting the diagnosis of DoC patients. With this aim, six studies transposed different CRS-R items in a BCI interface. Due to the importance of visual items in the differential diagnosis between VS/UWS and MCS (Wannez et al., 2018), they are the most frequently addressed in the above-mentioned studies: visual fixation and visual pursuit allow to differentiate between VS/UWS and MCS- and object recognition discriminates between the MCS– and MCS+. Visual fixation has a high prognostic value, but its high variability among DoC patients and during the same day makes it one of the main factors contributing to the high misdiagnosis rate (Candelieri et al., 2011; Cortese et al., 2015). Moreover, the evaluation of visual abilities is highly influenced by the subjectivity of the examiner, and this may lead to misinterpretations (Majerus et al., 2005). In all the studies that proposed a “BCI-based CRS assessment”, a varying proportion of patients (range: 30.8–78.6%) reached a classification accuracy above the chance level in the BCI assessment, while they did not reach the corresponding score in the CRS-R assessment. These promising results eventually support the contribution of the BCI in disambiguating confounding behavioral responses, by directly measuring brain activity. However, one study (Xiao et al., 2018a) also revealed that 2 patients who were able to perform visual fixation in the CRS-R assessment did not reach a significant classification accuracy in the BCI assessment, thus drawing attention to the importance of being careful when interpreting negative BCI results in this population of patients, considering the possibility for the BCI of failing in detecting signs of consciousness. All these studies, except for Wang et al. (2017), validated the systems on healthy subjects; furthermore, they found that part of the patients had a consistent response to both the BCI and the CRS assessment, contributing to validate the reliability of the BCI.

The study of Murovec et al. (2020) addressed the importance of a BCI-based repeated assessment procedure for DoC patients. A significant classification accuracy with a P300-based vibrotactile BCI was reached by only 10 out of 20 DoC patients during the first session, and by all the patients during their best run. This issue was already recognized with respect to the behavioral assessment of consciousness (Wannez et al., 2017). Indeed, a single BCI session may be affected by fluctuations in responsiveness (Piarulli et al., 2016), which may prevent patients from accomplishing the active task necessary to operate the BCI. Furthermore, the authors also investigated any possible training-effect with the BCI on the level of consciousness (i.e., CRS-R score). Even if a significant difference between the CRS-R score pre- and post-training was found, the absence of a control group prevented from drawing definitive conclusions regarding the BCI for rehabilitation purposes in DoC patients and highlights the need for further investigations.

Two studies aimed at investigating the correlation between BCI performance and the clinical outcome at 3 (Pan et al., 2020) and 6 months (Spataro et al., 2018), both finding a significant correlation between the presence of significant classification accuracy and a better prognosis with respect to patients who did not reach a classification accuracy above the chance level. Despite still preliminary, these results are highly promising since the evaluation of the prognosis, together with the assessment of consciousness, is an essential issue to address. Indeed, information about diagnosis and prognosis have a fundamental role in decisions about life sustaining therapies and in the economic management of the patients (Giacino et al., 2018). The BCI can provide a support to a more precise evaluation.

Regarding the possibility to use the BCI as an alternative communication channel, results are still preliminary. BCIs for communication mainly relied on the P300, both alone and in combination with another brain signal (i.e., hybrid control). A single study aimed at testing a MI-based BCI for communication, but none of the patients reached a classification accuracy sufficient for communication during the assessment phase (Chatelle et al., 2018). Among the other 6 studies testing communication, some significative results may be found in Wang et al. (2017) and Guger et al. (2018). In the first one, 2 VS/UWS patients managed to use the vibrotactile P300-based BCI to answer some personal questions. In the second one, the 61.5% of the patients reached a classification accuracy above the chance level during the communication assessment with an audiovisual P300-based BCI, indicative of the ability to use the BCI for communication purposes. An encouraging result can also be found in Huang et al. (2021b), in which 42.9% of the patients had a classification accuracy sufficient for communication. Annen et al. (2018) and Chatelle et al. (2018), who tested a vibrotactile P300-based BCI for communication, did not obtain consistent results, since none of the patients tested reached a satisfying and functional communication rate. Furthermore, Chatelle et al. (2018) tested the BCI for communication only on a LIS patient, since none of DoC patients had an accuracy rate sufficient for communication during the assessment phase. A similar pattern of results can be found in Lulé et al. (2013): only one LIS patient, and none of the DoC patients, reached a classification accuracy suitable for communication (60%) with an auditory P300-based BCI.

### Classification algorithms

With respect to the classification algorithms, we did not find a systematic correspondence between applications and specific classification algorithms, since the different algorithms are used indiscriminately across different applications. The SVM and LDA are the most common classifiers adopted in brain disorder research, because they are fast, easy to implement, and furnish interpretable results, managing to find a compromise between good classification performance and moderate complexity of the solution (Mechelli and Viera, 2019). Specifically, the LDA is mainly applied in studies using the *mindBEAGLE*, which is a commercial BCI consisting in an integrated system with tools for stimulus presentation and data recordings and analysis. The high frequency of SVM-based classifiers among the studies included in this review, may be due to some advantages that it can provide. Indeed, its performance is well balanced both in re-substitution and generalization, it allows to reduce overfitting and to eventually approach non-linearly separable classes. However, new approaches, such as the random forest and eXtreme Gradient Boosting algorithms (Paul et al., 2018), are becoming more widespread and they could be more efficient from a methodological perspective (Mechelli and Viera, 2019). Furthermore, some recent studies (Lee et al., 2022) adopted deep-learning approaches to classify the level of consciousness. A next step could be the integration of knowledge from the deep-learning field in the BCI domain, when managing DoC patients.

### Limits

Despite being very promising, these results are highly variegated, and many aspects may contribute to this variability. Indeed, many factors may affect the performance with the BCI and restrict its application in this target population; therefore, absence of results cannot be interpreted as absence of consciousness. Most DoC patients lack oculomotor control, which reduces the feasibility of visual information in BCI applications (Kalmar and Giacino, 2005), and they may face severe cognitive deficits, such as aphasia (Schnakers et al., 2015a; Formisano et al., 2019c), which compromises the ability to control a BCI. Furthermore, as previously underlined, fluctuations in responsiveness may affect BCI performance. Therefore, an active task, usually required when implementing a BCI, could be an advantage but also a limitation. Responsiveness to an active task is a stronger and more reliable index of consciousness: a positive response to an active task may be considered as the ability of command following. On the other hand, requiring patients to accomplish an active task, may lead to the same issues of a behavioral assessment. Indeed, an active task requires the ability to comprehend the instructions and to consequently perform it (e.g., to count the rare stimulus, to imagine the hand movement); comprehension problems derived from aphasia may prevent from the proper use of a BCI. These issues preclude from discriminating whether the absence of consistent results is due to the lack of awareness or to cognitive deficits that prevent from accomplishing the task properly. Another aspect to consider is a well-known phenomenon in the BCI field, the “*BCI illiteracy*” (Blankertz et al., 2010), which refers to the inability of some potential users to control a BCI (about 15–30%; Thompson, 2019). This phenomenon has been observed across different brain signals adopted to control a BCI (e.g., P300, SMRs). However, with respect to DoC patients, it is difficult to disambiguate if a lack of results may be explained in terms of BCI illiteracy or in terms of lack of consciousness.

Another problem when interpreting results from BCI is related to a lack of standardization of terms and tools of analysis in the BCI field, which may prevent the generalization and comparison of results among studies. Indeed, while there is agreement on the necessity of a standardization among the BCI community, it is still difficult to achieve this goal. The possibility to share and merge datasets from different research groups would allow more powerful statistical results (Singh et al., 2021). This is particularly relevant when the target population is represented by patients with DoC: effects in these patients may be very small due to all the factors previously mentioned. Therefore, standardizing and merging data from different studies may help in drawing more definitive conclusions. A key point in this sense is the lack of standardization with respect to the chance level calculation. Indeed, it is appropriate to calculate an adjusted chance level, considering the number of trials and subjects; however, the majority of the studies used a theoretical chance level, which does not consider the real distribution of the data. Noirhomme et al. (2014) tested the hypothesis that a permutation test, adjusted on the real distribution of data, is more appropriate than a binomial test in the definition of the chance level significance. This issue may lead to a misinterpretation in the significance of the BCI accuracies, contributing to create difficulties in the comparison of different results.

### Conclusions and recommendations for future implementation

In conclusion, despite still preliminary, almost all the studies reported positive results in a proportion of DoC patients, thus validating the importance and feasibility of BCI with these potential users. BCIs have the advantage to directly measure brain activity, thus overcoming problems related to behavioral assessment, and the subjectivity of the examiner. This could help in detecting patients with covert consciousness and promote functional communication. Although is necessary to face several limitations, some important key points can be inferred from this review, which should be accounted for future implementation of a BCI for DoC patients:

- The P300 is the most frequent brain signal employed to develop a BCI. The P300 has two main advantages, especially with respect to DoC patients: it requires a short calibration period, and it could be elicited in different sensory modalities, which is a fundamental aspect with this target population.
- As already suggested, it is necessary to implement a BCI application for diagnostic purposes before any other application (Annen et al., 2020a). Indeed, it is mandatory to assess the level of responsiveness, necessary to accomplish more complex tasks. A passive BCI could be a promising instrument in this sense (Han et al., 2022).
- A multimodal assessment (i.e., the combination of different sensory modalities) is encouraged in patients with DoC, also when implementing a BCI-based assessment; many studies proposed a h-BCI or BCI based of multiple sensory modalities (e.g., audiovisual BCIs). Furthermore, it would be recommended to adapt the BCI to the characteristics of the specific user, in order to employ the most appropriate sensory channel with respect to the patient's brain lesions.
- A repeated assessment could lead to better results. As for the behavioral assessment, also BCI assessment may be influenced by fluctuations in responsiveness, especially when patients are required to accomplish an active task.

## Data availability statement

The raw data supporting the conclusions of this article will be made available by the authors, without undue reservation.

## Author contributions

VG contributed to the conception and design of this systematic review, data collection and interpretation of results, and drafted and revised the manuscript. AR contributed to the conception of this review, interpretation of results, and drafting and revising of the manuscript. IQ and MD'I contributed to the drafting, interpretation of results, and revised the manuscript. FS, PA, SS, RF, FC, and DM participated in interpreting data and revising the manuscript. All authors contributed to the article and approved the submitted version.

## Funding

This present work was supported by the Italian Ministry of Health (MoH) under the Programme - Giovani Ricercatori 2019 (Project Number: GR-2019-12369824) and it was partially supported by grant from the European Union's Horizon 2020 Research and Innovation Program Under the Marie Skłodowska-Curie Grant Agreement No. 778234 - DoCMA Project.

## Conflict of interest

PA was employed by BrainSigns srl.

The remaining authors declare that the research was conducted in the absence of any commercial or financial relationships that could be construed as a potential conflict of interest.

## Publisher's note

All claims expressed in this article are solely those of the authors and do not necessarily represent those of their affiliated organizations, or those of the publisher, the editors and the reviewers. Any product that may be evaluated in this article, or claim that may be made by its manufacturer, is not guaranteed or endorsed by the publisher.
