# Supplementary material for: EEG-based Brain-Computer Interfaces for people with Disorders of Consciousness: Features and applications. A systematic review
Source: Front Hum Neurosci. 2022 Dec 5;16:1040816. doi: 10.3389/fnhum.2022.1040816 (PMC9760911; doi:10.3389/fnhum.2022.1040816)
Supplement: Supplementary file 1 [file Table_1.DOCX]

Supplementary Material

**Table 1**. The table summarize all the characteristics extracted from the 27 studies included in the review. *Acc: accuracy; CFS: Command Following Score; Ch: channels; CRS-R: coma recovery scale-revised; DAD: discriminant analysis with diagonal quadratic function; DE: differential entropy; GUI: graphical user interface; h-BCI: hybrid BCI; HC: healthy control; ISI: inter-stimulus interval; Knn: k-nearest neighbor; LDA: linear discriminant analysis; LIS: locked-in syndrome; LPC: late positive component; MCS: minimally conscious state; MI: motor imagery; MA: motor action; REW-NPLS: Recursive Exponentially Weighted N-way Partial Least Squares; SD: stimulus duration; SF: stimulus frequency; SMRs: sensorimotor rhythms; SOA: Stimulus Onset Asynchrony; SSVEP: steady state visual evoked potential; SVM: support vector machine; SWLDA: step-wise linear discriminant analysis; T: target; NT: non-target; VEP: visual evoked potential; VS/UWS: vegetative state/unresponsive wakefulness syndrome.*

| **STUDY** | **APPLICATION** | **PARTICIPANTS and ASSESSMENT** | **EEG FEATURE** | **EEG CHANNELS** | **CLASSIFICATION** | **PARADIGM** | | **BCI PERFORMANCE** |
| --- | --- | --- | --- | --- | --- | --- | --- | --- |
|  |  |  |  |  |  | **TASK** | **STIMULATION** |  |
| **Annen et al., 2018** | ASSESSMENT | 4 MCS 8 VS/UWS (*CRS-R*) | P300 | N=8  Fz, Cz, C3, C4, CPz, CP1, CP2, Pz | LDA | active vibrotactile oddball | stimuli: 2 tactors SD = 30ms SF = 225Hz ISI = 270ms n° stimuli = 480 paradigm duration = 2.4min T:NT = 1:7 | 1 MCS: classification acc > 70% in the vibrotactile paradigm with 2 and 3 stimuli |
|  | COMMUNICATION | 1 MCS |  |  |  | active vibrotactile oddball | stimuli: 3 tactors task: 6 autobiographic questions to answer within 30s n° trials training phase = 4 n° stimuli per training trial = 90 standard, 15 T, 15 NT SD = 30ms SF = 225Hz ISI = 270ms | 1/6 correct answer |
| **Annen et al., 2020b** | ASSESSMENT | 12 HC 2 EMCS 23 MCS 15 VS/UWS (*CRS-R*) | P300 | N=8  FCz, C3, Cz, C4, CP1, CPz, CP2, Pz | LDA | active auditory oddball | stimuli: 2 auditory stimuli (T, NT) n° stimuli = 480 n° T stimuli = 60 SD = 100ms ISI = 900ms | 25% patients significative acc only in one paradigm  (online chance level: 23%, binomial test offline chance level: permutation test, p<.05) |
|  |  |  |  |  |  | active vibrotactile oddball | stimuli: 2 tactors  paradigm duration = 2.4min T:NT = 1.25:8.75 SD = 30ms  SF = 225Hz ISI = 270ms |  |
| **Chatelle et al., 2018** | ASSESSMENT | 10 HC 1 LIS 4 MCS 1 VS/UWS 4 coma (*CRS-R*) | P300 | N=8  Fz, C3, Cz, C4, CP1, CPz, CP2, Pz | LDA | active auditory oddball | 2 auditory stimuli (T, NT) sequences n° = 4  stimuli n° = 15 T, 105 NT per sequence SD = 100ms  ISI = 900ms | - 3/10 patients (2 coma, 1 VS/UWS) significant classification acc in the auditory paradigm - 3/10 patients (1 VS/UWS, 1 MCS, 1 LIS) significant classification acc in the vibrotactile paradigm - no patients with significant classification acc in the MI |
|  |  |  |  |  |  | active vibrotactile oddball | stimuli: 2 and 3 tactors sequences n° = 4  stimuli n° = 15 T, 105 NT per sequence SD = 110ms  SF = 250Hz  ISI = 300ms |  |
|  |  |  | SMRs | N=16  Fz, C3, Cz, C4, CP1, CPz, CP2, Pz, FC3, FC4, C5, C1, C2, C6, CP3, CP4 | LDA | MI | task: open/close hand trials n° = 30 left, 30 right hand trial duration = 6s |  |
|  | COMMUNICATION | 8 HC 1 LIS (*CRS-R*) | P300 | N=8  Fz, C3, Cz, C4, CP1, CPz, CP2, Pz | LDA | active vibrotactile oddball | stimuli: 3 tactors assessment paradigm acc > 60% questions n° = 2 sequence duration = 38s | 1 LIS patient: 1/2 correct answer |
|  |  | 6 HC | SMRs | N=16  Fz, C3, Cz, C4, CP1, CPz, CP2, Pz, FC3, FC4, C5, C1, C2, C6, CP3, CP4 | LDA | MI | task: open/close hand assessment paradigm acc > 60% questions n° = 2 sequence duration = 38s |  |
| **Coyle et al., 2015** | ASSESSMENT | 4 MCS (*CRS-R*) | SMRs | Phase 1: N=3 Phase 2: N=16 | LDA | MI | task: move the hand/foot toe  phase 1: INITIAL BCI ASSESSMENT blocks n° = 6 (alternate right hand/toe blocks) trials n° = 15 per block 15 beep tones synchronized with a cue arrow on the screen during execution  inter-block break = 1-2min  phase 2: TRAINING with auditory/visual feedback trials n° = 60 per repetition repetitions n° = 1-2 per session repetition duration= 8min sessions n° ≤ 8 1 session per day possible feedback: basket-ball, spaceship games, broadband noise, musical sample | - BCI can supplement awareness assessment - auditory feedback more suitable than visual one  (chance level: 50% to 60%, nonparametric Wilcoxon signed-rank test) |
| **Eliseyev et al., 2021** | ASSESSMENT | 5 HC 14 conscious 4 unconscious  (*CFS*) | SMRs | N=21  C3, C4, O1, O2, A1, A2, Cz, F3, F4, F7, F8, Fz, Fp1, Fp2, P3, P4, Pz, T7, T8, P7, P8 | REW-NPLS | MA | task: open/close hand calibration session n° = 1-2 calibration session duration = 5min paradigm session n° = 1 paradigm session duration = 10min inter-session break duration = 5min  calibration: 5min (1 or 2 sessions) testing stage: 10min | 5 conscious patients control the BCI |
| **Guger et al., 2017** | ASSESSMENT | 5 DoC  3 LIS | P300 | N=16  FC3, FCz, FC4, C5, C3, C1, Cz, C2, C4, C6, CP3, CP1, CPz, CP2, CP4, Pz | LDA | Active auditory oddball | ISI: 900ms  T:NT=1:7 (=60:420)  SF= 1000Hz (T), 800Hz (NT)  Paradigm duration: 7.3min | 7/8 patients acc > 80% |
|  |  |  |  |  |  | Active vibrotactile oddball | 2 tactors  SD= 100ms (40ms pause)  T:NT=1 :8  Paradigm duration: 2.5min  3 tactors  T :NT:NT=1 :1 :8 |  |
|  |  |  | SMRs |  |  | MI | Task: imagine left/right hand movement  SD= 4s (0.5-2s pause)  60 trials  Paradigm duration= 9 min |  |
|  | COMMUNICATION | 5 DoC  3 LIS | P300 | N=16  FC3, FCz, FC4, C5, C3, C1, Cz, C2, C4, C6, CP3, CP1, CPz, CP2, CP4, Pz | LDA | Active vibrotactile oddball | 3 tactors  SD= 100ms (40ms pause)  T :NT:NT=1 :1 :8  Paradigm duration: 2.5min | 3 patiemts (1 DoC, 2 LIS): efficient communication |
|  |  |  | SMRs |  |  | MI | Task: imagine left/right hand movement  SD= 4s (0.5-2s pause)  60 trials  Paradigm duration= 9 min | 1 LIS : efficient communication |
| **Guger et al., 2018** | ASSESSMENT | 12 VS/UWS (*CRS-R*) | P300 | N=8  Fz, C3, Cz, C4, CP1, CPz, CP2, Pz | LDA | active vibrotactile oddball | stimuli: 2 and 3 tactors SD = 100ms SF = 80Hz n° stimuli 2 tactors run = 30 groups of 8 (120 left, 120 right) n° stimuli 3 tactors run = 30 groups of 8 (30 left, 30 right, 180 distractors) | - 7/12 VS/UWS discriminable brain response (2 tactors oddball) - 5/12 VS/UWS discriminable brain response (3 tactors oddball; 2 VS/UWS acc >70%)  (chance level: 23%, binomial test) |
|  | COMMUNICATION |  |  |  |  | active vibrotactile oddball | stimuli: 3 tactors SD = 100ms SF = 80Hz n° stimuli per question = 120 stimuli, 38s T:NT = 1:7 (only if assessment paradigm accuracy > 70%) | 2 VS/UWS tested for communication (acc>80%): - 4/5 correct answers - 6/10 and 7/10 correct answers |
| **Holler et al., 2013** | ASSESSMENT | 22 HC 5 MCS 9 VS/UWS (*CRS-R*) | SMRs | N=32 recorded  3 EOG, 2 re-references, Fp1, Fp2, F3, F4, C3, C4, P3, P4, O1, O2, F7, F8, T7, T8, P7, P8, Fz, Cz, Pz, FC1, FC2, CP1, CP2, FC5, FC6, CP5, CP6  N=21 processed  F3, F4, C3, C4, P3, P4, O1, O2, F7, F8, Fz, Cz, Pz, FC1, FC2, CP1, CP2, FC5, FC6, CP5, CP6 | DADF knn SVM, linear kernel | MI | task: open/close hand conditions = 3 (resting, movement, imagery) resting instruction = 6s movement instruction = 6.5s  imagery instruction = 9.5s time after each instruction = 5s n° trials = 24 per condition | Coherences showed the best reliability among HC and patients |
| **Huang et al., 2021 (a)** | ASSESSMENT | 10 HC 1 EMCS 6 MCS 1 VS/UWS (*CRS-R*) | DE  (delta, theta, alpha, beta, gamma bands) | N=32 | SVM, linear kernel | emotion recognition  (passive) | GUI: emotional videoclip calibration: 10 trials experiment: 10 trials  sessions: 5 (1 per day)  trial phases:  1. hint about negative/positive emotion (5 s) 2. clip (30s)  3. feedback of recognized emotion (5 s) 4. rest (5 s) | 3/8 patients (2 MCS, 1 EMCS) online significant classification acc  (significance level: 64%, chi-Quadro test) |
| **Huang et al., 2021 (b)** | COMMUNICATION | 11 HC 4 MCS 3 VS/UWS (*CRS-R*) | h-BCI: P300, SSVEP | N=9  CPz,P7, P3, Pz, P4, P8, O1, Oz, O2 | SVM, linear kernel | active visual oddball | GUI: 2 visual stimuli (YES/NO) blocks n° = 5 (1 per day) trials n° = 10 (1 per block)  trial phases: 1. instruction (8s),  2. stimulation (10s) -> flickering and flashing (right square flickering= 7.5Hz, left square flickering= 6Hz, flash duration = 200ms, flash ISI = 800ms, flashes n° = 10, 5 right, 5 left) 3. feedback (4s) 4. rest (2s) | 3 MCS/7 patients significant classification acc  (significance level: 64%, chi-Quadro test) |
| **Li et al., 2015** | ASSESSMENT | 4 HC 2 EMCS 3 MCS 6 VS/UWS (*CRS-R*) | h-BCI: P300, SSVEP | N=10 recorded  Fz, Cz, P7, P3, Pz, P4, P8, O1, Oz, O2  N=8 processed  P7, P3, Pz, P4, P8, O1, Oz, O2 | SVM | active visual oddball | GUI: 2 visual stimuli (one-digit numbers) calibration run: 10 trials 3 run (number recognition, number comparison, mental calculation) blocks n° = 5 per run  trials n° = 10 per block  trial phases:  1. instruction (8s) 2. stimulation -> right square flickering = 7.5Hz, left square flickering = 6 Hz, flash duration = 200ms, flash ISI= 800ms, flash n°= 5 right, 5 left (10s) 3. feedback (4s) 4. rest (2s) | - 5/11 patients significant classification acc in 2/3 tasks (2 VS/UWS, 2 MCS, 1 EMCS) - 3/5 patients (1 VS/UWS, 1 MCS, 1 ECMS) significant classification acc in 3/3 tasks  (significance level: 64%, chi-Quadro test) |
| **Lule et al., 2013** | ASSESSMENT | 16 HC 2 LIS 13 MCS 3 VS/UWS (*CRS-R*) | P300 | N=16  F3, Fz, F4, T7, T8, C3, Cz, C4, Cp3, Cp4, P3, Pz, P4, PO7, PO8, Oz | SWLDA | active auditory oddball | stimuli: 4 auditory stimuli (YES, NO, GO, STOP) run 1 (calibration) = 4 trial run 2 (test) = 12 trial (HC), 10 (patients) SD = 400ms ISI = 600ms trial duration = 1min | 2/18 patients (1 MCS, 1 LIS) P300 response to target  (chance level: 60%, chi-Quadro test) |
|  | COMMUNICATION |  |  |  |  |  |  | 1 LIS: acc 60% |
| **Murovec et al., 2020** | ASSESSMENT | 6 HC 9 MCS 11 VS/UWS (*CRS-R*) | P300 | N=8  FCz, C3, Cz, C4, CP1, CPz, CP2, Pz | Fisher LDA | active vibrotactile oddball | stimuli: 3 tactors  n° calibration run = 1 n° sessions = 10 (1 per day) n° run = 8-12 per session run duration = 2.5min n° stimuli per run = 480  T:NT= 1:7 SD = 100ms ISI = 100ms | - 10/20 patients significant classification acc in the first run - 20/20 patients significant classification acc in the best run  (chance level: 23%) |
|  | REHABILITATION |  |  |  |  |  |  | significant difference in the CRS score pre-post training |
| **Pan et al., 2014** | ASSESSMENT | 4 HC 1 LIS 3 MCS 4 VS/UWS (*CRS-R*) | h-BCI: P300, SSVEP | N=10  Fz, Cz, P7, P3, Pz, P4, P8, O1, Oz, O2 | SVM | active visual oddball | GUI: 2 visual stimuli (personal/unfamiliar photo) calibration run: 10 trials 3 runs (own photo/unfamiliar photo/own or unfamiliar photo) blocks n° = 5 per run  trials n° = 10 per block  trial phases:  1. instruction (8s) 2. stimulation -> right square flickering = 7.5Hz, left square flickering = 6Hz, flash duration = 200ms, flash ISI = 800ms, flash n° = 5 right, 5 left (10s) 3. feedback (4s)  4. rest (2s) | - 5/8 patients (2 VS/UWS, 2 MCS, 1 LIS) significant classification acc in the first run - 3/5 patients (1 VS/UWS, 1 MCS, 1 LIS) significant classification acc in run 2 and 3  (significance level: 64%, chi-Quadro test) |
| **Pan et al., 2018** | ASSESSMENT | 8 HC 5 MCS 3 VS/UWS (*CRS-R*) | P300 | N=30 | SVM | active audiovisual oddball | GUI: 2 emotional audiovisual clips trials n° = 20 (calibration run) blocks n° = 5 (evaluation run; 1 per day)  trials n° = 10 per block stimuli n° = 8 laughing clips, 8 crying clips SD = 1.400ms  ISI = random [500-1.200]ms  trial phases: 1. instructions (10s) 2. stimulation (36s) 3. feedback (4s)  4. break (10s) | 3/8 patients (1 VS/UWS, 2 MCS) significant classification acc (significance level: 64%, chi-Quadro test) |
| **Pan et al., 2020** | PROGNOSIS | 8 HC 33 MCS 45 VS/UWS (*CRS-R*) | h-BCI: P300, SSVEP | N=30 | SVM, linear kernel | active visual oddball  active audiovisual oddball | GUI: 2 stimuli-> 1) photographs (visual), 2) numbers (visual), audiovisual numbers calibration = 10 trials  blocks n° = 5 (1 per day) trials n° = 10 per block  trial phases:  1. instruction (focus on your own/stranger's photograph or on a number 8s) 2. stimulation: right square flickering = 7.5Hz, left square flickering = 6Hz, flash duration = 200ms, flash ISI = 800ms, flash n° = 5 right, 5 left (10s) 3. feedback (4s) 4. rest (10s) | patients with significant classification acc had statistically better outcome than patients without significant classification acc  (chance level: 64%, chi-Quadro test) |
|  | ASSESSMENT |  |  |  |  |  |  | 44% patients significant classification acc  (significance level: 64%, chi-Quadro test) |
| **Pokorny et al., 2013** | ASSESSMENT | 10 HC 12 MCS (*CRS-R*) | P300 | N=15 recorded (HC)  F3, Fz, F4, T7, C5, Cz, C6, T8, TP7, CP5, CP6, TP8, P3, Pz, P4  N=9 recorded (MCS) F3, Fz, F4, C3, Cz, C4, P3, Pz, P4)  N=3 processed  Fz, Cz, Pz | SWLDA | active auditory oddball | stimuli: 2 tone streams with deviant tone at random positions (low tone stream (LTS), high tone stream (HTS))  tones duration = 60ms  LTS -> standard LTS frequency = 396Hz, deviant LTS frequency = 297Hz, ISI = 300ms, deviant tones = 10%  HTS -> standard HTS frequency = 1900Hz, deviant HTS frequency = 2640Hz, ISI = 600ms, deviant tones = 20%  silent gap ( _ ) = 150ms tone stream pattern HC: LHL_LHL runs n° = 8 (HC), 4 (MCS) trials n° = 10 per run (HC), 5 (MCS) trial duration = instruction (3-8s), tone streams (30s), break (8-12s) | none of the patients had a classification acc (>70%) |
| **Spataro et al., 2018** | ASSESSMENT | 6 HC 13 VS/UWS (*CRS-R*) | P300 | N=8  Fz, C3, Cz, C4, CP1, CPz, CP2, Pz | LDA | active vibrotactile oddball | stimuli: 2 and 3 tactors runs n°= 1 oddball with 2 tactors + 1 with 3 tactors per session stimuli n°= 8 per sequence sequences n° = 30 per trial trials n° = 4 SD = 100ms 2 tactors: NT:T= 7:1  run duration = 2.5 min 3 tactors: left wrist:right wrist:shoulder = 1:1:6 run duration = 3 min | 3/12 VS/UWS significant classification acc  (threshold of responsiveness: acc ≥ 50%) |
|  | PROGNOSIS |  |  |  |  |  |  | significant correlation between BCI performance and 6-months CRS follow-up |
| **Spataro et al., 2022** | ASSESSMENT | 2 VS/UWS  14 MCS | P300 | N=8  FCz, Cz, CPz, Pz, C3, C4, CP1, CP2 | LDA | Active vibrotactile oddball | stimuli: 3 tactors  session= 4 run  run= 4 trials block  trial block= 30 trials  trial= 8 stimuli  T:NT= 1:7  run duration = 2.5 min SOA=400ms SD = 100ms | 1^st^ week assessment:  - CRS-R: 14/16 patients no command following  - BCI: 8/14 patients command following  3^rd^ week assessment:  - 1 patient no command following in CRS-R/command following in BCI  - 1 patient already command following in BCI, command following in CRS-R  - No changes in other patients  7^th^ week assessment:  - Final diagnosis (CRS-R): 7 VS/UWS, 3MCS, 6 MCS+  - 10 patients (4 VS/UWS) command following in BCI  (chance accuracy: 12.5%) |
| **Wang et al., 2015** | ASSESSMENT | 10 HC 4 MCS 3 VS/UWS (*CRS-R*) | P300 | N=30 | SVM | active audiovisual oddball | GUI: 2 audiovisual stimuli (numbers) SD = 300ms  ISI = random [700-1500]ms HC (visual, auditory, audiovisual): calibration trials n° = 10  experiment trials n° = 30   patient (audiovisual): calibration trials n° = 10 experiment blocks n° = 5 (1 per day) trials n° = 10 per block | 5/7 patients (1 VS/UWS, 4 MCS) significant classification acc  (significance level: 61.4%, binomial test) |
| **Wang et al., 2017** | ASSESSMENT | 5 MCS  8 VS/UWS (*CRS-R*) | P300 | N=30 | SVM | active audiovisual oddball | GUI: 2 audiovisual stimuli (YES/NO) calibration block n°= 1  blocks n° = 5 (1 per day)  trials n° = 12 per block SD = 300ms ISI = random [700-1500]ms  trial phases:  1. instruction (20s) 2. stimulation (28s) -> 2 buttons, audiovisual stimulus of each button presented 5 times 3. feedback (4s) 4. rest (10s) | 8/13 patients (4 VS/UWS, 4 MCS) significant classification acc: - 1 MCS responsive CRS/BCI - 7 patients unresponsive to CRS/responsive to BCI  (significance level: 60.4%, binomial test) |
|  | COMMUNICATION |  |  |  |  |  |  |  |
| **Wang et al., 2019** | ASSESSMENT | 8 HC 1 LIS 7 MCS 5 VS/UWS (*CRS-R*) | P300 | N=30 | SVM | active audiovisual oddball | GUI: 2 audiovisual stimuli (3D-stereo objects) calibration: 10 trials experiment: 4 blocks  trials n°= 10 per block  trial phases: 1. instruction (object to focus on 6s) 2. stimulation (random flashes of 2 objects with simultaneous correspondent spoken word 28s) 3. feedback (4s) 4. rest (≥ 10s) | - 6/13 patients (2 VS/UWS, 3 MCS, 1 LIS) significant classification accuracy: - none of the patients had a score=5 at the CRS  (significance level: 65.5%, chi-quadro test) |
| **Xiao et al., 2016** | ASSESSMENT | 5 HC 1 EMCS 6 MCS 14 VS/UWS (*CRS-R*) | P300 MMN | N=32 recorded N=4 processed (Fz, FCz, Cz, CPz) | peak detection algorithm | passive auditory oddball | stimuli: 2 auditory stimuli (T, NT) trials n° = 30 (HC), 20 (DoC) rounds n° = 5 per trial, 10 (DoC) stimuli n° = 4 standard, 1 deviant per round trial duration = 29s (HC), 49s (DoC) online feedback duration = 3s inter-trial break= [1-10]s | - 14/19 patients (9 VS/UWS, 4 MCS, 1 EMCS) auditory startle response to both CRS/BCI - 3/19 patients (VS/UWS) auditory startle response only with the BCI  (chance level: 40%, chi-Quadro test) |
| **Xiao et al., 2018 (a)** | ASSESSMENT | 5 HC 1 LIS  1 EMCS 5 MCS 8 VS/UWS  (*CRS-R*) | P300 | N=32 | SVM, linear kernel | active visual oddball | GUI: 4 visual stimuli (1 T) SD = 100ms ISI = 100ms n° stimuli = 4 per round (800ms), 10 rounds per trial (1s) n° trials per session = 10 calibration, 10 online n° sessions = 2 4 buttons + 1 moving ball, 2.5s for the ball to reach one of the buttons | 4/15 patients (1 VS/UWS, 2 MCS, 1 LIS) significant classification acc: - 3/4 (2 MCS; 1 LIS) response to both CRS/BCI - 1 VS/UWS response only with the BCI  2/15 patients (1 MCS, 1 EMCS) response to the CRS, no with the BCI  (significance level: 45%, chi-Quadro test) |
| **Xiao et al., 2018 (b)** | ASSESSMENT | 5 HC 1 LIS 1 EMCS 6 MCS 6 VS/UWS (*CRS-R*) | P300 N170 motion-onset VEP | N=32 | SVM | active visual oddball | GUI: 4 visual stimuli (1 T) sessions n° =2  blocks n° = 2 per session (1 calibration, 1 training) trials n°= 10 per block  trial phases: 1. instruction (7s)  2. stimulation (10 repetition, 8s) -> 4 button flash 10 times in a random order (100ms, ISI = 100ms): 3 unfamiliar faces, 1 subject's photo moving from the center to the initial position 3. feedback (4s)  4. rest (≥ 10s) | 11/14 patients significant classification acc: - 4 patients (2 MCS; 1 EMCS, 1 LIS) responsive to the CRS/BCI - 7 patients (4 VS/UWS, 3 MCS) responsive only with the BCI  (significance level: 45%, chi-Quadro test) |
| **Xiao et al., 2022** | ASSESSMENT | 18 HC 10 VS/UWS 8 MCS (*CRS-R*) | P300 MMN | N=30 | SVM | active audiovisual oddball | stimuli: 2 visual stimuli (T + auditory stimulation, NT) session: 1 calibration (10 trials), 1 online mode (10 trials) sessions n° = 2 trial n° = 40  trail phases: 1. instruction (7s) 2. auditory cue (3s) 3. 10 stimulations (35s) -> 2 buttons flashed in random order, ISI = random [900, 1000, 1100, 1200, 1300, 1400]ms 4. feedback (2s) 5. rest | - 4 MCS responsive to the CRS/BCI - 7 patients (5 VS/UWS, 2 MCS) responsive only with the BCI  (chance level: 67%, binomial test) |
| **Xie et al., 2018** | ASSESSMENT | 10 HC 3 MCS 5 VS/UWS (*CRS-R*) | P300 N400 LPC | N=32 | SVM, linear kernel | active audiovisual oddball | GUI: 2 semantically congruent/incongruent audiovisual one-digit numbers calibration: 10 trials (per session) experiment: 40 trial (per session) SD = 300ms, ISI =700ms, trial duration= 8 rounds, 4 stimuli per round | 3/8 patients (1 VS/UWS, 2 MCS) significant classification accuracy  (significance level: 37.3%, binomial test) |
